# Proteolysis of Virulence Regulator ToxR Is Associated with Entry of *Vibrio cholerae* into a Dormant State

**DOI:** 10.1371/journal.pgen.1005145

**Published:** 2015-04-07

**Authors:** Salvador Almagro-Moreno, Tae K. Kim, Karen Skorupski, Ronald K. Taylor

**Affiliations:** Department of Microbiology and Immunology, Geisel School of Medicine at Dartmouth, Hanover, New Hampshire, United States of America; Universidad de Sevilla, SPAIN

## Abstract

*Vibrio cholerae* O1 is a natural inhabitant of aquatic environments and causes the diarrheal disease, cholera. Two of its primary virulence regulators, TcpP and ToxR, are localized in the inner membrane. TcpP is encoded on the *Vibrio* Pathogenicity Island (VPI), a horizontally acquired mobile genetic element, and functions primarily in virulence gene regulation. TcpP has been shown to undergo regulated intramembrane proteolysis (RIP) in response to environmental conditions that are unfavorable for virulence gene expression. ToxR is encoded in the ancestral genome and is present in non-pathogenic strains of *V. cholerae*, indicating it has roles outside of the human host. In this study, we show that ToxR undergoes RIP in *V. cholerae* in response to nutrient limitation at alkaline pH, a condition that occurs during the stationary phase of growth. This process involves the site-2 protease RseP (YaeL), and is dependent upon the RpoE-mediated periplasmic stress response, as deletion mutants for the genes encoding these two proteins cannot proteolyze ToxR under nutrient limitation at alkaline pH. We determined that the loss of ToxR, genetically or by proteolysis, is associated with entry of *V. cholerae* into a dormant state in which the bacterium is normally found in the aquatic environment called viable but nonculturable (VBNC). Strains that can proteolyze ToxR, or do not encode it, lose culturability, experience a change in morphology associated with cells in VBNC, yet remain viable under nutrient limitation at alkaline pH. On the other hand, mutant strains that cannot proteolyze ToxR remain culturable and maintain the morphology of cells in an active state of growth. Overall, our findings provide a link between the proteolysis of a virulence regulator and the entry of a pathogen into an environmentally persistent state.

## Author Summary

Non-obligate bacterial pathogens must alter their gene expression profiles when transitioning between environments. *Vibrio cholerae* is a natural inhabitant of aquatic ecosystems and the etiological agent of the severe diarrheal disease, cholera. Its virulence gene regulation is controlled by a complex transcriptional cascade involving a membrane-localized regulator termed ToxR. Here we show that ToxR undergoes proteolysis under nutrient limitation at alkaline pH and this loss is associated with the entry of *V*. *cholerae* into a dormant state, similar to that found in its natural environment between epidemics. Thus, to our knowledge, we provide the first evidence of a link between the proteolysis of a virulence regulator and the entry of a bacterial pathogen into an environmentally persistent state.

## Introduction

The ability of microorganisms to alter their gene expression profiles when transitioning between environments is fundamental for their survival. *Vibrio cholerae* O1 is a non-obligate pathogen that switches between the human host, where it colonizes the intestinal epithelium, and the aquatic environment where it is found as free-living or attached to biotic and abiotic surfaces [1–4]. Upon entry of *V*. *cholerae* into the human host, the expression of its two major virulence factors is induced: the toxin co-regulated pilus (TCP), an essential intestinal colonization factor [5], and cholera toxin (CT), responsible for the diarrhea associated with the disease [5,6]. The expression of these factors is coordinately regulated at the transcriptional level by a virulence cascade involving a number of regulatory proteins [7]. Central to this cascade is the cooperation between two pairs of membrane-localized transcriptional regulators, TcpPH, encoded on the *Vibrio* Pathogenicity Island (VPI) [8], and ToxRS, encoded in the ancestral genome and also present in non-pathogenic isolates of *V*. *cholerae*. These findings indicate that ToxR has roles outside of the human host. Both TcpPH and ToxRS are required to activate the transcription of the master virulence regulator, ToxT [9–12]. ToxT, also encoded on the VPI, directly activates the expression of TCP and CT, as well as other genes [7,13,14].

Once intestinal colonization and proliferation have taken place, *V*. *cholerae* downregulates the expression of the virulence cascade [8,15,16]. In the classical biotype of *V*. *cholerae*, termination of virulence is mediated by proteolysis of the major virulence activator ToxT [9–12,17] as well as by the regulated intramembrane proteolysis (RIP) of TcpP [18]. RIP is a widely distributed mechanism in both prokaryotes and eukaroytes for responding to extracellular signals and stresses [19–21]. The initial proteolytic event in the RIP of TcpP is catalyzed by a currently unidentified site-1 protease, resulting in a TcpP species that is further degraded by the site-2 metalloprotease RseP (YaeL) [18]. The most widely studied example of RIP activates the σ^E^-dependent envelope stress response [22,23]. This process involves RseA, a membrane localized anti-σ factor that arrests RpoE. In response to envelope stress, RpoE is released for interaction with RNA polymerase by a RIP event in which RseA is sequentially cleaved by the serine protease DegS at a periplasmic site, and then by RseP at an intramembrane site [24–26].

The downregulation of virulence gene expression late in the infection process coincides with the upregulation of genes that promote detachment of bacteria from the mucosal surface of the intestine and that enhance the survival of *V*. *cholerae* when it returns to the environment [27–29]. Regulatory systems involved in controlling the expression of these genes include the quorum sensing regulator HapR [27], the stationary phase alternative sigma factor RpoS [28], and the VarS/VarA two component system [29]. Once in the environment, biofilm formation and entry into a dormant state known as viable but nonculturable (VBNC) or conditionally viable environmental cells (CVEC), play crucial roles in the survival of *V*. *cholerae* by facilitating its environmental persistence within aquatic habitats during periods between epidemics [4,30–33]. CVEC are clumps of dormant cells embedded in a biofilm matrix that can be recovered using enriched culturing techniques [31]. Quorum sensing has been implicated in the regulation of CVEC [31,34]. Nonetheless, the molecular mechanisms governing entry into VBNC remain to be elucidated.

Microarray analysis has revealed that ToxR influences the expression of more than 150 genes in *V*. *cholerae* [35]. Besides virulence, regulated genes include those involved in cellular transport, energy metabolism, motility, and iron uptake. In addition, ToxR reciprocally regulates the expression of two outer membrane porins, OmpU and OmpT, in response to the nutritional status of the cell [36–38]. Furthermore, unlike *tcpP*, *toxR* expression is not altered under conditions that favor the maximal expression of virulence genes in the laboratory [39,40]. In this study, we show that under nutrient limitation at alkaline pH, ToxR levels decrease by a RIP mediated event. This process is dependent upon the metalloprotease RseP, which appears to function as a site-2 protease, and the σ^E^-mediated periplasmic stress response, which likely provides a site-1 protease that contributes to the cleavage of ToxR prior to RseP. We determined that the loss of ToxR, either by proteolysis or genetically, is associated with the entry of *V*. *cholerae* into a dormant, nonculturable state that is similar to the VBNC state observed in the natural environment.

## Results

### ToxR levels are reduced in response to nutrient limitation at alkaline pH

**Fig 1:**
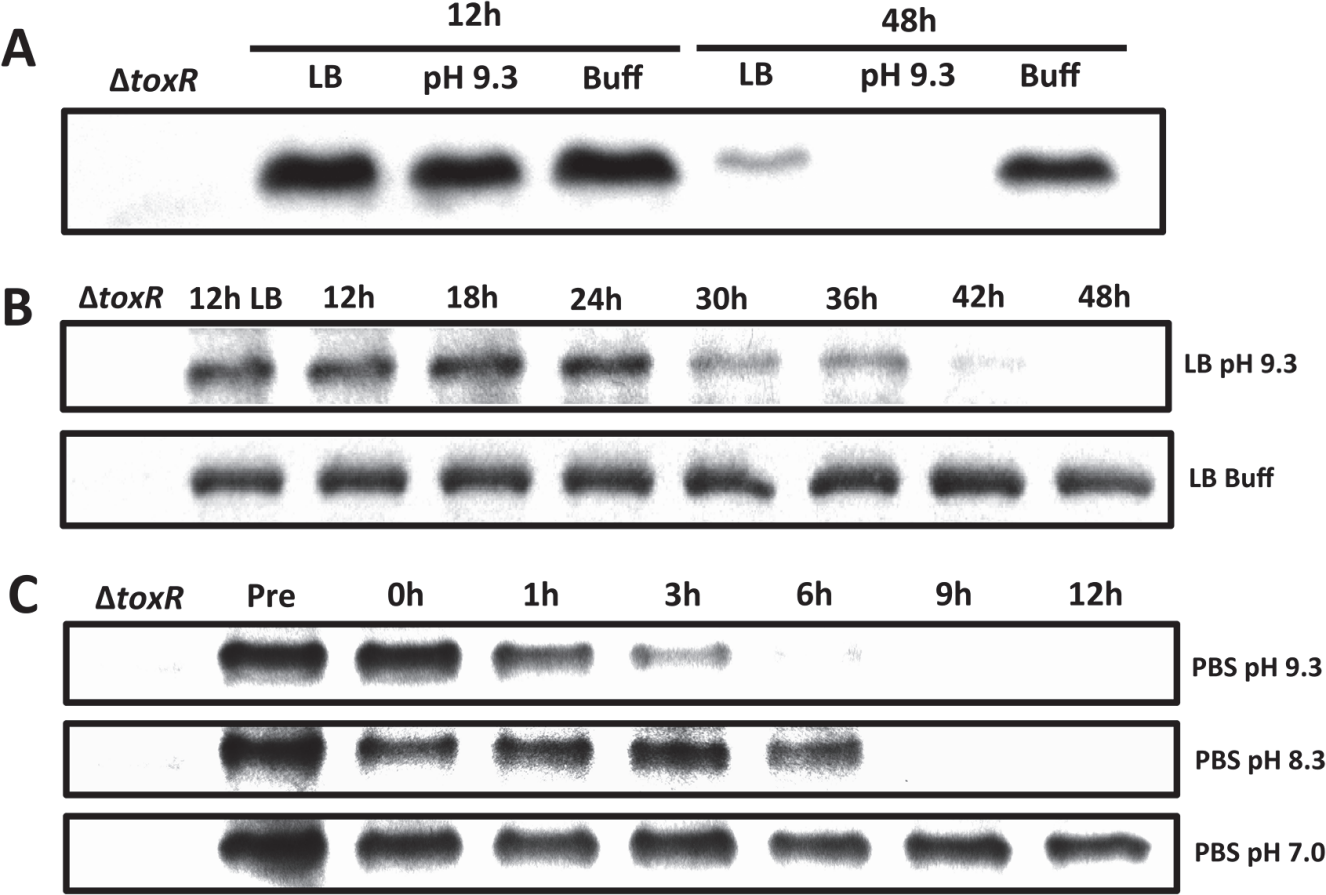
Proteolysis of ToxR during late stationary phase at alkaline pH. **(A)** ToxR immunoblot of O395 wild-type or Δ*toxR* grown for either 12 or 48 hours in LB starting pH 7.0 unbuffered (LB), LB starting pH 9.3 unbuffered (pH 9.3), or LB buffered to pH 7.0 with 100 mM HEPES (Buff). **(B)** ToxR immunoblots of O395 wild-type grown at different time points in LB starting pH 9.3 unbuffered (pH 9.3), or LB buffered to pH 7.0 with 100 mM HEPES (Buff). **(C)** ToxR immunoblots of O395 wild-type grown overnight in LB starting pH 7.0 unbuffered at 37°C, pelleted, and resuspended in phosphate buffered saline (PBS) at pH 7.0, pH 8.3, or pH 9.3 for 12 hours.

The expression of *ompU* is activated by ToxR in nutrient abundant environments, such as in rich medium [36] or in the host [41], whereas the expression of *ompT* is derepressed in nutrient limited conditions, such as in minimal medium [38] or during the late stationary phase of growth [37]. Since the levels of ToxR increase in the presence of nutrients to facilitate *ompU* expression [38], we hypothesized that the levels of ToxR would decrease during the growth of *V*. *cholerae* in late stationary phase to facilitate *ompT* expression. To test this, wild-type classical biotype strain O395 was grown in LB medium (starting pH 7.0, unbuffered) for 12 and 48 hours. As shown in Fig 1A, the levels of ToxR after 48 hours were significantly reduced compared to the 12 hour culture. The final pH of the 48 hour culture was determined to be 9.2–9.3, indicating that the medium became alkaline during growth. To determine whether alkaline pH contributed to the loss of ToxR after 48 hours, *V*. *cholerae* was grown for 12 and 48 hours in LB medium with a starting pH of 9.3 (unbuffered) and also in LB medium buffered at pH 7.0 with 100 mM HEPES. As shown in Fig 1A, ToxR was undetectable in cultures grown for 48 hours in LB at pH 9.3, whereas its levels remained high at this pH when grown for only 12 hours. This indicates that, in LB, ToxR appears to start being proteolyzed once the cultures reach both alkaline pH and nutrient limitation. When the medium was buffered at pH 7.0, ToxR levels remained high even after 48 hours (Fig 1A). Thus, in late stationary phase, ToxR levels significantly decrease after 48 hours due to an increase in the pH. As a control we examined the stability of a ToxR-unrelated protein, GbpA, and found that these conditions did not affect the stability of GbpA (S1A Fig) [42]. We then determined the dynamics of ToxR proteolysis by measuring ToxR protein levels between 12 hours and 48 hours post-inoculation in LB pH 9.3 at 6 hour intervals (Fig 1B). We found that ToxR proteolysis begins around 24 hours of growth at LB pH 9.3 (Fig 1B). ToxR becomes undetectable between 42 and 48 hours of culture (Fig 1B).

The observation that ToxR levels start decreasing after 24 hours at pH 9.3 (Fig 1B) suggests that nutrient limitation associated with late stationary phase might influence the levels of ToxR in stationary phase. To assess this, overnight cultures of O395 grown in LB at 37°C were pelleted and resuspended in phosphate buffered saline (PBS) at pH 7.0 or pH 9.3 for 12 hours. As shown in Fig 1C, ToxR levels decreased after only 3 hours in response to nutrient limitation at pH 9.3, whereas they did not significantly decrease in response to nutrient limitation at neutral pH. When overnight cultures were transferred to PBS at pH 8.3, the levels of ToxR started decreasing between 6 and 9 hours (Fig 1C). These results indicate that in late stationary phase, ToxR levels decrease in response to nutrient limitation at alkaline pH.

### Proteolysis of ToxR during late stationary phase at alkaline pH occurs in an RseP and σ^E^-dependent manner

**Fig 2:**
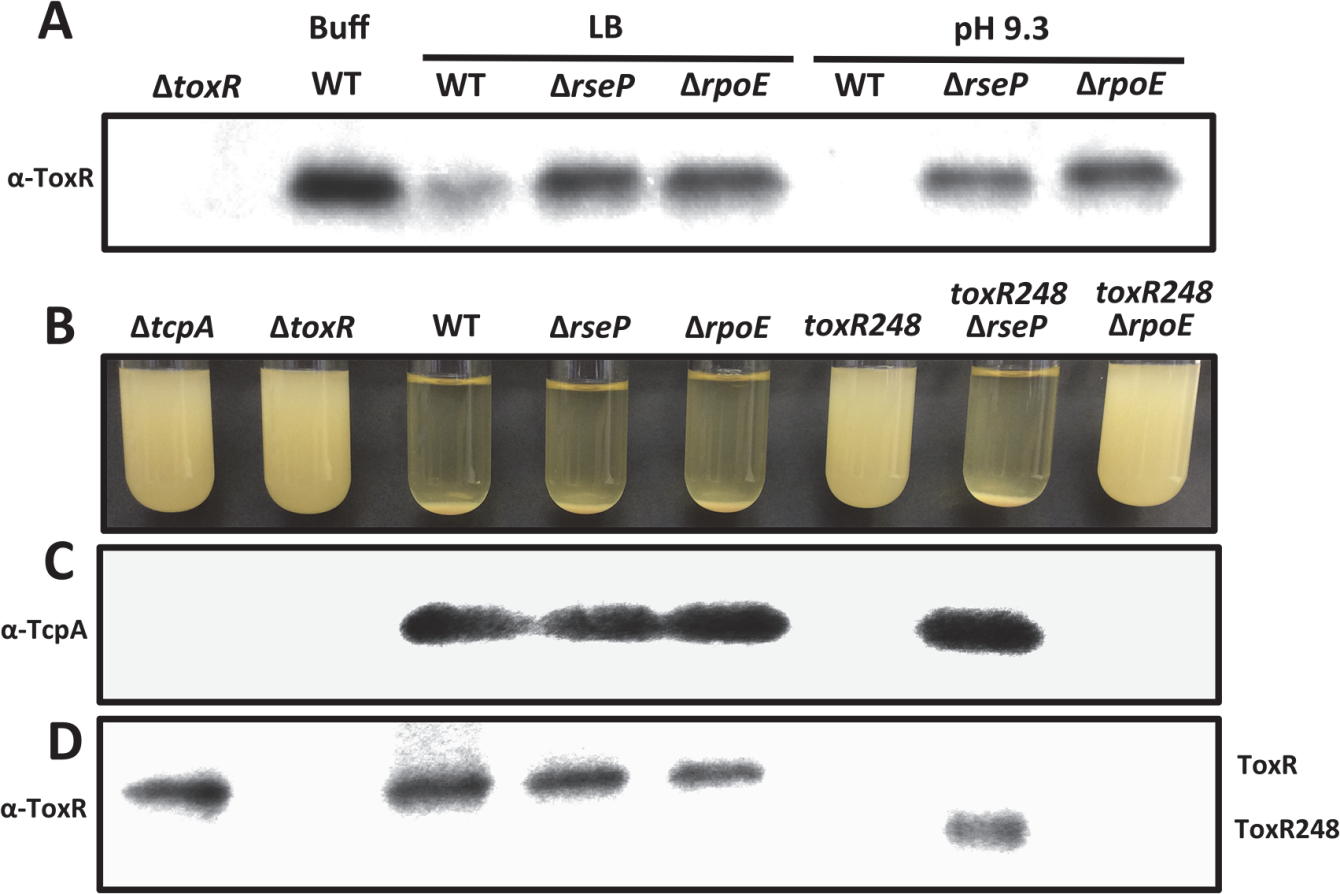
Proteolysis of ToxR is RseP and RpoE-dependent. **(A)** ToxR immunoblot of cultures of O395 wild-type or Δ*toxR*, Δ*rseP* or Δ*rpoE* grown for 48 hours in LB starting pH 7.0 unbuffered (LB), LB starting pH 9.3 unbuffered (pH 9.3), or LB buffered to pH 7.0 with 100 mM HEPES (Buff). **(B)** Autoagglutination of O395 Δ*tcpA*, Δ*toxR*, wild-type (WT), Δ*rseP*, Δ*rpoE*, *toxR248*, *toxR248*Δ*rseP* and *toxR248*Δ*rpoE* grown under inducing conditions (LB starting pH 6.5, 30°C) for 15 hours. Autoagglutination can be visualized as a pellet at the bottom of the tube. **(C)** TcpA and **(D)** ToxR immunoblots of the cultures in (B).

The levels of TcpP have been shown to be controlled by RIP through the site-2 protease RseP [18]. We determined whether the levels of ToxR in response to nutrient limitation at alkaline pH may also be influenced by proteolysis through RseP. As shown in Fig 2A, ToxR was largely undetectable in strain O395 after growth for 48 hours in LB with a starting pH of 7.0 or 9.3, whereas the levels of ToxR in the Δ*rseP* mutant under these conditions were similar to wild-type grown in LB buffered at pH 7.0 with 100 mM HEPES. This finding indicates that RseP is involved in the proteolysis of ToxR during late stationary phase at alkaline pH, possibly functioning as a site-2 protease.

The ToxR activated porin OmpU functions as an outer membrane sensor responding to damage induced by antimicrobial peptides and triggers activation of the σ^E^-dependent envelope stress response by promoting DegS-mediated cleavage of RseA [43]. We hypothesized that OmpU may also function as a sensor responding to alkaline pH during late stationary phase and induce the activation of the σ^E^ pathway, ultimately leading to the proteolysis of ToxR. To address this, we assessed the levels of ToxR in a Δ*rpoE* mutant after growth for 48 hours in LB with a starting pH of 7.0 or 9.3. As shown in Fig 2A, the levels of ToxR in the Δ*rpoE* mutant under these conditions were similar to the Δ*rseP* mutant. These findings indicate that both RseP and RpoE play a role in the proteolysis of ToxR during late stationary phase at alkaline pH.

Although the proteolysis of ToxR in late stationary phase at alkaline pH depends upon the σ^E^ pathway, this process is either independent of the porin OmpU, or OmpU is insufficient to trigger the proteolysis of ToxR since an *ompU* mutant did not restore ToxR levels under this condition (S1 Table). To further investigate the role of RpoE in the proteolysis of ToxR, we cloned *rpoE* into an expression vector, pBAD22, and tested whether ectopic overexpression of *rpoE* affected the stability of ToxR (S1B Fig). We found that ectopic expression of *rpoE* does not trigger the proteolysis of ToxR, as ToxR was detectable at every time point we measured (S1B Fig). In addition, we tested the effect of conditions that induce the σ^E^ pathway on the stability of ToxR (S1C Fig) [43,44]. We determined that growth in 3% ethanol or exposure to P2 antimicrobial peptide did not induce proteolysis of ToxR (S1C Fig). Thus, it appears that the σ^E^ pathway alone is not sufficient to induce the proteolytic cascade that culminates with the RIP of ToxR. It is possible that a second pathway might act in conjunction with the σ^E^ pathway to orchestrate the proteolysis of ToxR, in a similar manner as the Cpx pathway and the σ^E^ pathway work in combination in order to monitor the cell envelope in *Escherichia coli* [45,46]. Interestingly, the Cpx two-component system, which partially overlaps with the σ^E^ pathway in the periplasmic stress response [46,47] does not play a role in the proteolysis of ToxR. In late stationary phase at alkaline pH, deletion of *cpxR*, a regulator of the pathway, did not restore the levels of ToxR (S1 Table).

### Periplasmic truncations of ToxR are proteolyzed in an RseP-dependent, σ^E^-independent manner

The involvement of σ^E^ in the proteolysis of ToxR raises the possibility that the role of this pathway is to provide a site-1 protease that contributes to the cleavage of its periplasmic domain. Since RseP is known to activate the σ^E^ pathway [25,26], the function of RseP in the proteolysis of ToxR may be to activate this pathway, or to also function directly as the site-2 protease of ToxR. To assess this, we generated periplasmic truncations of ToxR that should bypass the requirement for a site-1 protease and assessed the roles of RseP and σ^E^ in the proteolysis of these truncations. If RseP functions indirectly in the proteolysis of ToxR the introduction of a Δ*rseP* mutation into strains carrying the truncations should have no influence on their stability. A periplasmic truncation was generated by introducing a stop codon in ToxR at position 249 (amber, *toxR248*). When cells are grown under inducing conditions the production of TCP allows for the formation of microcolonies, clusters of bacterial cells tethered together; this process is dependent on the production of the major pilin subunit TcpA [48]. Due to their size, microcolonies flocculate and form a pellet in a phenomenon known as autoagglutination [5,48]. As shown in Fig 2B, the strain carrying *toxR248* failed to autoagglutinate, and neither TcpA nor ToxR could be detected by immunoblot (Fig 2C and2D) indicating that the truncated protein is proteolyzed. However, introduction of Δ*rseP* into the *toxR248* strain restored autoagglutination (Fig 2B), TcpA production (Fig 2C) and produced an intermediate species of ToxR (Fig 2D) indicating that RseP influences the stability of the truncated ToxR protein. In contrast, a Δ*rpoE* mutation in *toxR248* failed to autoagglutinate (Fig 2B) and did not stabilize the truncated ToxR protein (Fig 2D). These findings indicate that RseP plays a direct role in the cleavage of ToxR when a portion of its periplasmic domain is missing, and suggest that the effect of the σ^E^ pathway occurs prior to site-2 proteolysis of ToxR by RseP.

### Culturability of *V*. *cholerae* decreases over time at alkaline pH

**Fig 3:**
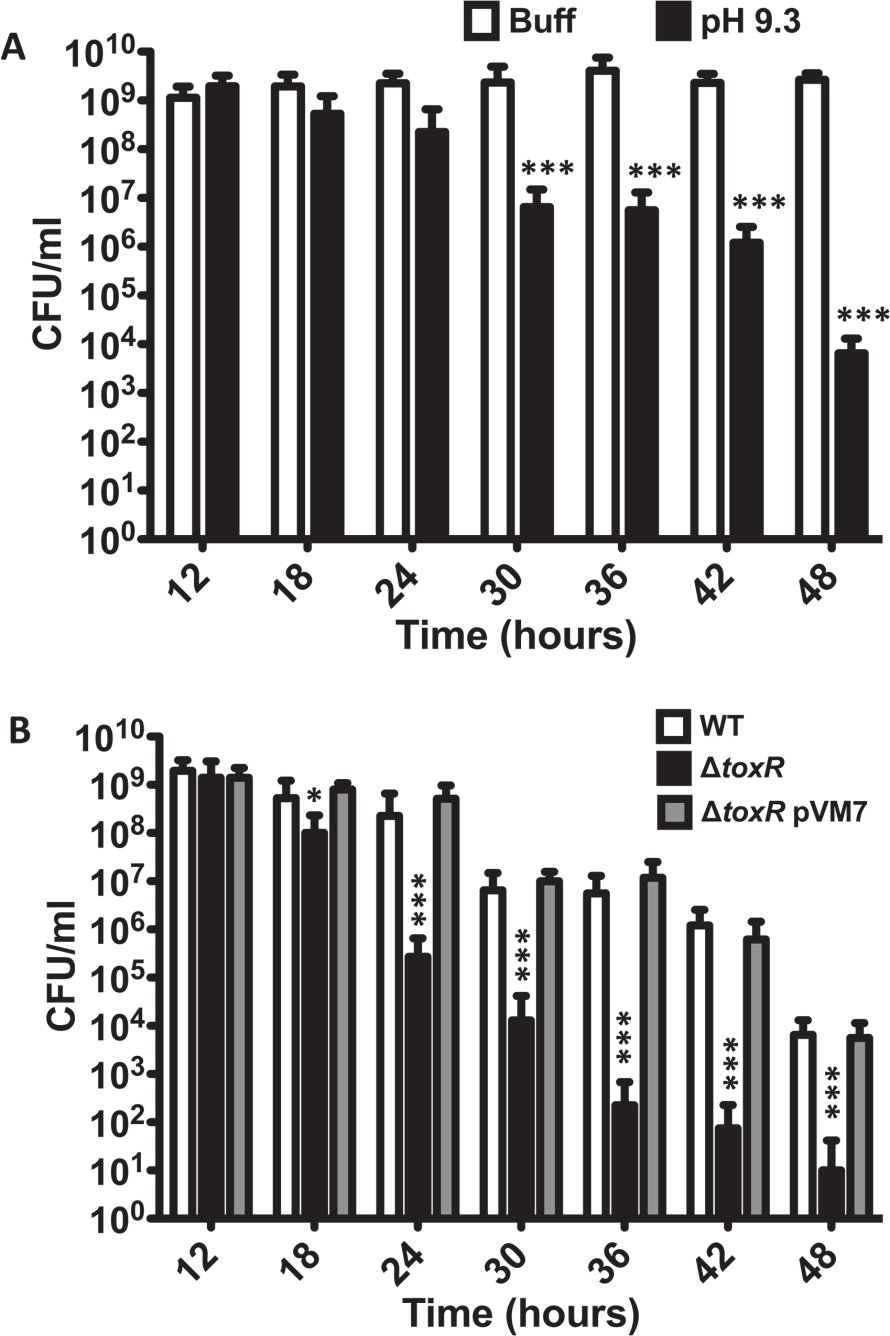
*V*. *cholerae* shows reduced culturability over time at alkaline pH. **(A)** CFU/ml of O395 wild-type strain grown at different time points in LB pH 7.0 with 100 mM HEPES (Buff), or LB starting pH 9.3 unbuffered (pH 9.3). The bars represent the mean of four independent experiments and the error bars indicate the standard deviation. Statistical comparisons were made using the student’s *t*-test and compare samples relative to 12h Buff. ***P < 0.0005. **(B)** CFU/ml of O395 wild-type (WT), Δ*toxR*, or Δ*toxR* pVM7 strains grown at different time points in LB starting pH 9.3 unbuffered. The bars represent the mean of four independent experiments and the error bars indicate the standard deviation. Statistical comparisons were made using the student’s *t*-test and compare samples relative to wild-type on LB pH 9.3 at that specific time point. *P < 0.05, ***P < 0.0005.

The proteolysis of ToxR under nutrient limitation at alkaline pH might provide an advantage to *V*. *cholerae* by preventing the expression of genes with roles in nutrient rich environments, such as OmpU, and promoting those with roles in nutrient poor conditions, such as OmpT [36–38]. Under conditions that are not conducive to active growth, such as nutrient limitation, *V*. *cholerae* is capable of entering a dormant, nonculturable state referred to as VBNC or CVEC that facilitates its survival and persistence [30,31,49–51]. We investigated whether the loss of ToxR is associated with the entry of *V*. *cholerae* into a nonculturable state. To assess this, the culturability of *V*. *cholerae* was measured by plating cultures of *V*. *cholerae* O395 between 12 and 48 hours after inoculation on LB medium with a starting pH of 9.3 and also in LB medium buffered at pH 7.0 with 100 mM HEPES at 6 hour intervals, and determining the colony forming units (CFUs) at each time point (Fig 3A). As shown in Fig 3A, the number of CFU/ml of O395 grown in LB 100 mM HEPES do not change over time whereas the number of CFU/ml of cultures grown on LB with a starting pH of 9.3 start getting reduced in some cultures around 24 hours of growth with a final CFU count nearly 5 logs lower than the cultures grown in LB buffered at pH 7.0 (Fig 3A). Interestingly, the culturability of *V*. *cholerae* is reduced more or less in parallel with the proteolysis of ToxR (Fig 1B). These results indicate that growth of *V*. *cholerae* to late stationary phase at alkaline pH, defined as ToxR proteolysis inducing (TPI) conditions, decreases the culturability of *V*. *cholerae*.

There appears to be a correlation between the proteolysis of ToxR and the loss of culturability of *V*. *cholerae* as the levels of the protein decrease at a similar time point as *V*. *cholerae* begins to lose culturability (Fig 1A and3A). We assessed whether a Δ*toxR* mutant strain would become nonculturable at a faster rate than the wild-type as, given that it will not produce ToxR from the beginning of the incubation period, it might lose culturability as soon as the suitable conditions are met. We found that, after 18 hours of growth on LB pH 9.3, when the nutrients are becoming scarce as the bacterium reaches mid-stationary phase, Δ*toxR* starts losing culturability (Fig 3B and S2B Fig). After 48 hours of growth at LB pH 9.3 the number of CFUs recovered ranged between 0 and 10^2^ (Fig 3B), indicating that Δ*toxR* becomes nonculturable at a faster rate than the wild-type strain when cultured on LB pH 9.3. On the other hand, the number of CFUs remained relatively constant when Δ*toxR* was cultured on LB buffered at pH 7.0 with 100mM HEPES, confirming that both alkaline pH and nutrient limitation are required for loss of culturability (S2 Fig). We also determined the culturability of a Δ*toxR* strain harboring a plasmid that constitutively expresses *toxR* (pVM7) [9]. The strain Δ*toxR* pVM7 shows a similar number of CFU/ml as the wild-type strain indicating that ectopic expression of ToxR recovers wild-type phenotype in the Δ*toxR* strain (Fig 3B).

Growth curves for both wild-type and Δ*toxR* on LB buffered and LB pH 9.3 show a similar pattern, indicating that there is no growth difference between the strains in these conditions (S2B Fig). Furthermore, a similar pattern, Δ*toxR* losing culturability faster than wild-type at pH 9.3, was found when the wild-type strain and Δ*toxR* were cultured on PBS pH 7 and PBS pH 9.3 (S3 Fig). The Δ*toxR* strain starts losing culturability as soon as the cultures are transferred to nutrient limiting conditions (PBS) at alkaline pH (S3B Fig).

### The loss of ToxR is required for loss of culturability of *V*. *cholerae* after 48 hours at alkaline pH

**Fig 4:**
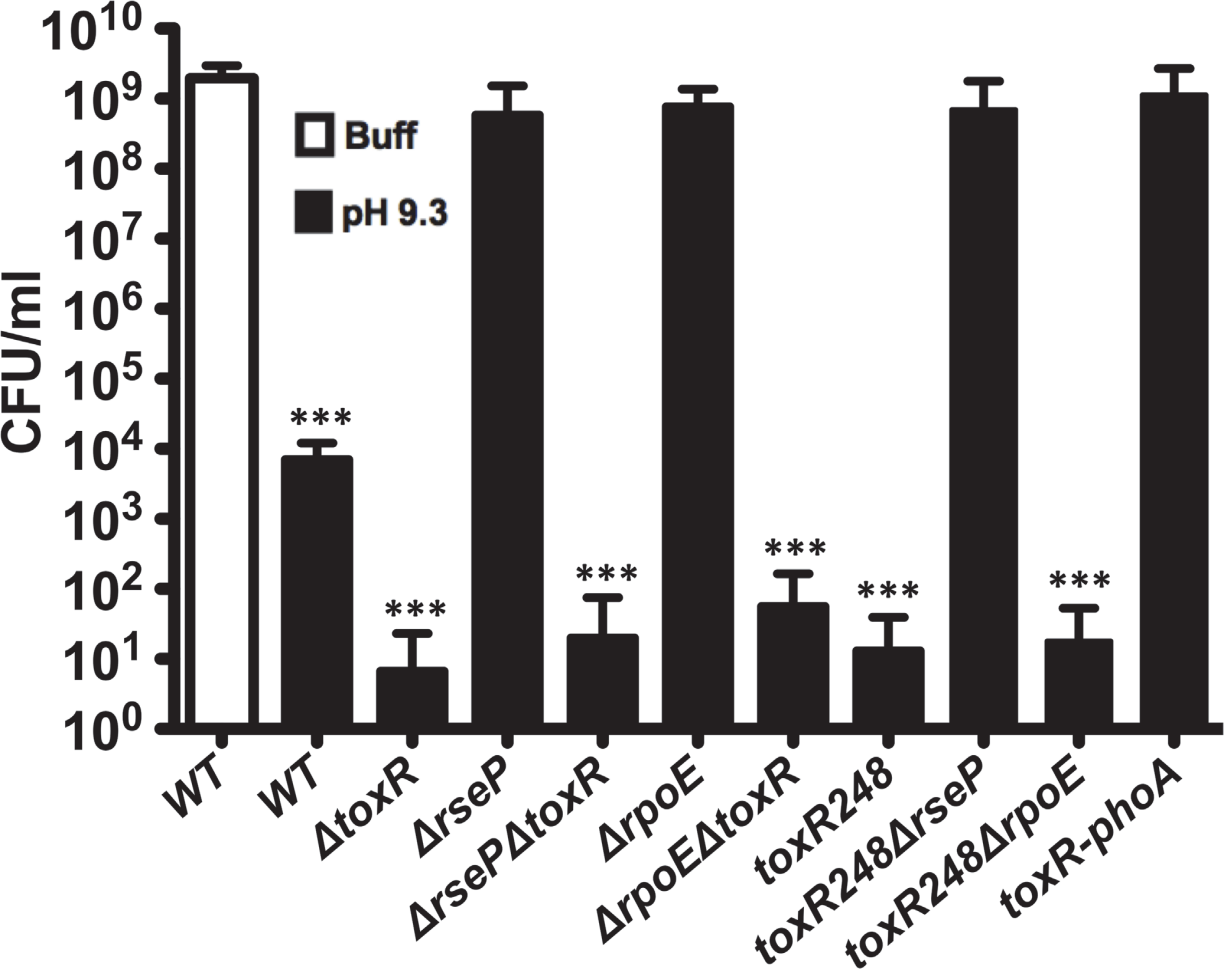
Reduction in culturability of *V*. *cholerae* depends on the loss of ToxR. CFU/ml of O395 wild-type (WT) and mutant strains in LB pH 7.0 with 100 mM HEPES (Buff), or LB starting pH 9.3 unbuffered (pH 9.3) for 48 hours. The bars represent the mean of at least four independent experiments and the error bars indicate the standard deviation. Statistical comparisons were made using the student’s *t*-test and compare samples relative to wild-type 48h Buff. ***P < 0.0005.

The same conditions that trigger the proteolysis of ToxR induce the loss of culturability of *V*. *cholerae* (Fig 1A and 3A). Additionally, a Δ*toxR* mutant becomes nonculturable at a significantly faster rate than the wild-type strain and its phenotype can be complemented by ectopic expression of ToxR (Fig 3B). To further investigate the relationship between ToxR and the culturability of *V*. *cholerae*, the CFUs under TPI conditions were determined for wild-type O395, Δ*toxR*, Δ*rseP* and Δ*rpoE* mutants. As shown in Fig 4, the number of CFU/ml for wild-type, which is able to proteolyze ToxR, was around 10^4^ and the Δ*toxR* mutant ranged between 0 and 10^2^. In contrast, the number of CFU/ml for Δ*rseP* and Δ*rpoE* mutants that are unable to proteolyze ToxR was approximately 10^9^ under this condition, similar to the number of CFUs recovered when the wild-type strain was grown on LB buffered at pH 7.0 with 100 mM HEPES (Fig 4). Introduction of the Δ*toxR* mutation into the Δ*rseP* and Δ*rpoE* backgrounds decreased the CFUs under TPI conditions similar to that of the Δ*toxR* mutant alone.

ToxR undergoes proteolysis in strains *toxR248* and *toxR248*Δ*rpoE* (Fig 2). We found that both strains show a highly reduced number of CFU/ml under TPI conditions, similar to the Δ*toxR* strain (Fig 4). The strain *toxR248*Δ*rseP* does not proteolyze ToxR, and an intermediate species of the regulator can be detected for this strain (Fig 2D). Consistently, the number of CFU/ml under TPI conditions for *toxR248*Δ*rseP* is similar to that of strains that cannot proteolyze ToxR such as wild-type under buffered conditions (Fig 4).

To further study the association between the loss of ToxR and loss of culturability of *V*. *cholerae*, we constructed a fusion strain, *toxR*-*phoA*, in which ToxR does not undergo proteolysis under TPI conditions (S3C Fig) [52]. The *toxR-phoA* fusion strain does not lose culturability under TPI conditions and show a similar number of CFUs as the strains that do not proteolyze ToxR (wild-type on LB buffered, Δ*rseP*, Δ*rpoE*, or *toxR248*Δ*rseP*) (Fig 4). Thus, the absence of ToxR, either by proteolysis or genetically, appears to be required for the loss of culturability of the strains.

### *V*. *cholerae* remains viable and displays an altered cellular morphology under TPI conditions

**Fig 5:**
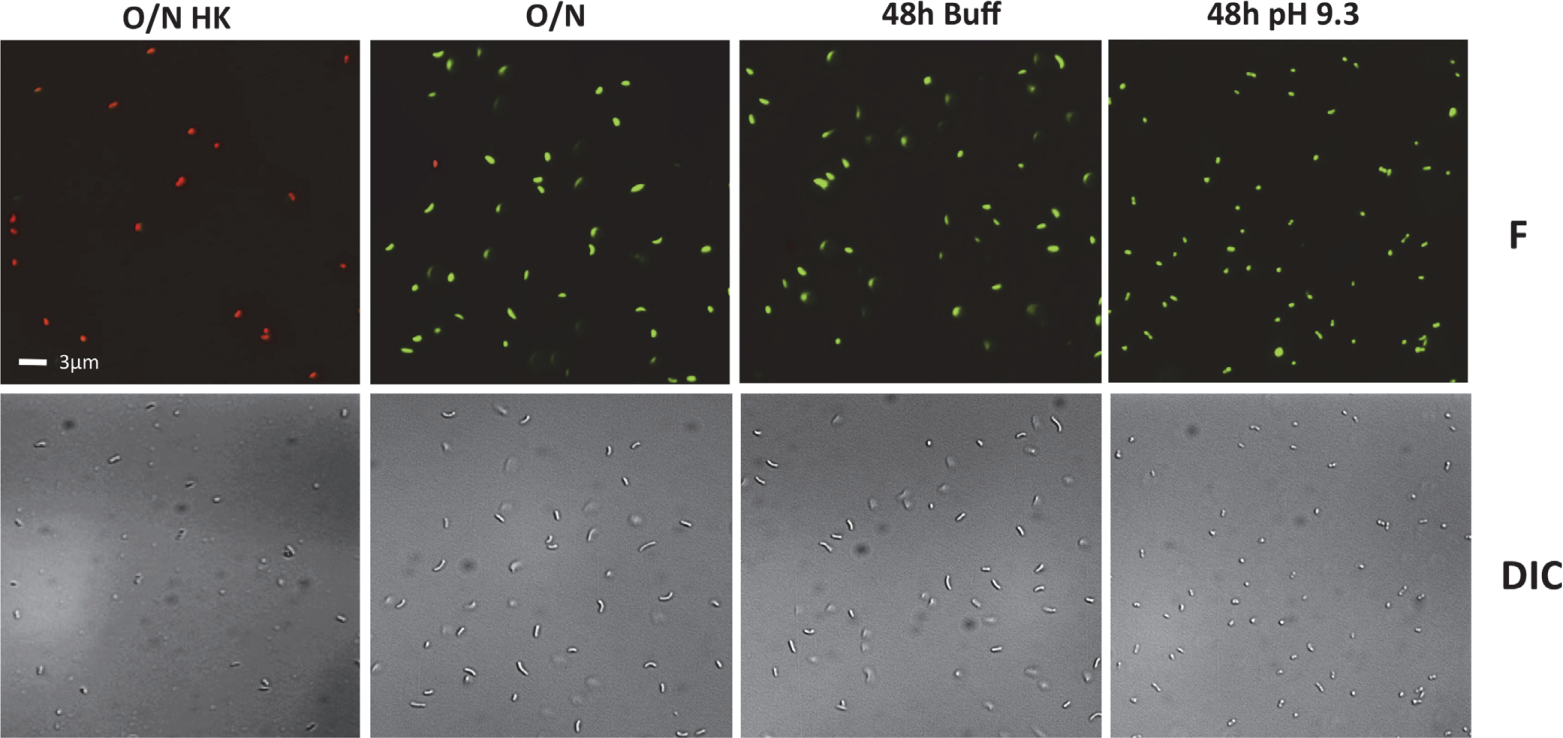
Viability and morphology of *V*. *cholerae* after 48 hours at alkaline pH. Fluorescent (F) and differential interference contrast (DIC) images of O395 wild-type grown in LB starting pH 7.0 unbuffered overnight and heat killed (O/N HK), LB starting pH 7.0 unbuffered overnight (O/N) as controls, 48 hours in LB buffered to pH 7.0 with 100 mM HEPES (48h Buff), and 48 hours in LB pH 9.3 unbuffered (48h pH 9.3).

To determine whether the decreased culturability of the wild-type and some of the mutants under TPI conditions is due to their entry into a dormant state and not cell death, the viability of the cells in the cultures in Fig 4 were examined using the LIVE/DEAD BacLight Bacterial Viability and Counting Kit. This kit allows for the discernment between viable cells (green) and dead cells (red) using fluorescent microscopy. The entry of *V*. *cholerae* into a dormant state is associated with loss of culturability (Fig 4), maintenance of viability (green cells), and a change in its morphology from elongated rods to round, coccoid-shaped cells [53,54]. Dead cells (O/N HK) can be seen as red under fluorescence (F), whereas cells from an overnight culture (O/N) are green and elongated (F and DIC) (Fig 5). We found that when grown in LB pH 7.0 buffered with 100 mM HEPES (48h Buff) the wild-type shows a morphology and viability similar to O/N (Fig 5), indicative of a culturable state. Under TPI conditions (48h pH 9.3), the cells remain viable and alive (green) and their morphology changes to a coccoid form, indicative of entry into a dormant state (Fig 5).

**Fig 6:**
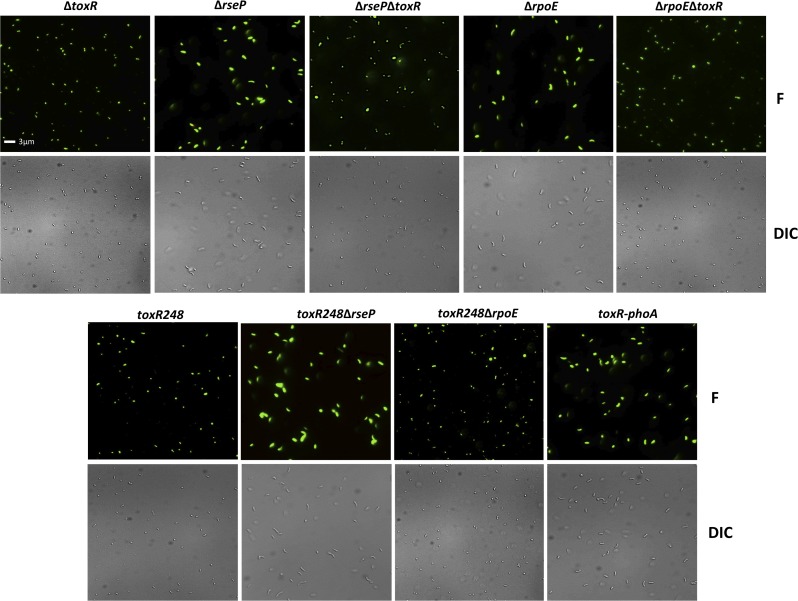
Viability and morphology of *V*. *cholerae* mutants after 48 hours at alkaline pH. Fluorescent (F) and differential interference contrast (DIC) images of O395 Δ*toxR*, Δ*rseP*, Δ*rseP*Δ*toxR*, Δ*rpoE* or Δ*rpoE*Δ*toxR*, *toxR248*, *toxR248*Δ*rseP*, *toxR248*Δ*rpoE*, and *toxR-phoA* grown for 48 hours in LB starting pH 9.3 (unbuffered). The cells were observed after treatment with the LIVE/DEAD BacLight Bacterial Viability and Counting Kit. Viable and culturable cells appear green and elongated; viable but dormant cells appear green and round; dead cells appear red and round.

The viability and morphology of the mutant strains tested in Fig 4 is consistent with their culturability (Fig 6). The strains that cannot proteolyze ToxR (Δ*rseP*, Δ*rpoE*, *toxR248*Δ*rseP*, and *toxR-phoA*) show a similar morphology and viability to cells in O/N cultures (Figs 5 and 6), whereas the mutants that do not encode ToxR (Δ*toxR*, Δ*rseP*Δ*toxR*, and Δ*rpoE*Δ*toxR*) or proteolyze it (*toxR248* and *toxR248*Δ*rpoE*) are viable and round (Fig 6). These findings indicate that in *V*. *cholerae* the loss of ToxR is associated with entry into a dormant state.

### The loss of ToxR is also associated with entry of *V*. *cholerae* El Tor biotype and *Vibrio parahaemolyticus* into a dormant state

*V*. *cholerae* is classified into more than 200 serogroups, however, only the O1 serogroup causes epidemic cholera. *V*. *cholerae* O1 is further classified into two biotypes, classical and El Tor. Strain O395, where previous studies regarding termination of virulence had been made, belongs to the classical biotype [17,18]. Strains of the El Tor biotype are the source of the current pandemic of cholera and were recently shown to enter VBNC differentially when compared with classical [55]. We determined the effect of TPI conditions in the El Tor biotype strain N16961. We found that severely reduced levels of ToxR in late stationary phase were also observed with the El Tor biotype strain N16961 (S4A Fig). In this strain, it was necessary to incubate for 72 hours at pH 9.3 to visualize complete loss of ToxR by immunoblot. For N16961, the number of CFU/ml of cultures grown for 72 hours in LB with a starting pH of 9.3 was over 4 logs lower than the number of CFU/ml of cultures grown in LB buffered at pH 7.0 with 100mM HEPES (Fig 4B). We also found that when grown in LB pH 7.0 buffered with 100 mM HEPES, N16961 remains viable (green) and elongated (S4C Fig), indicative of a culturable state whereas under TPI conditions, the cells are viable and round (S4C Fig), indicative of entry into a dormant state.

**Fig 7:**
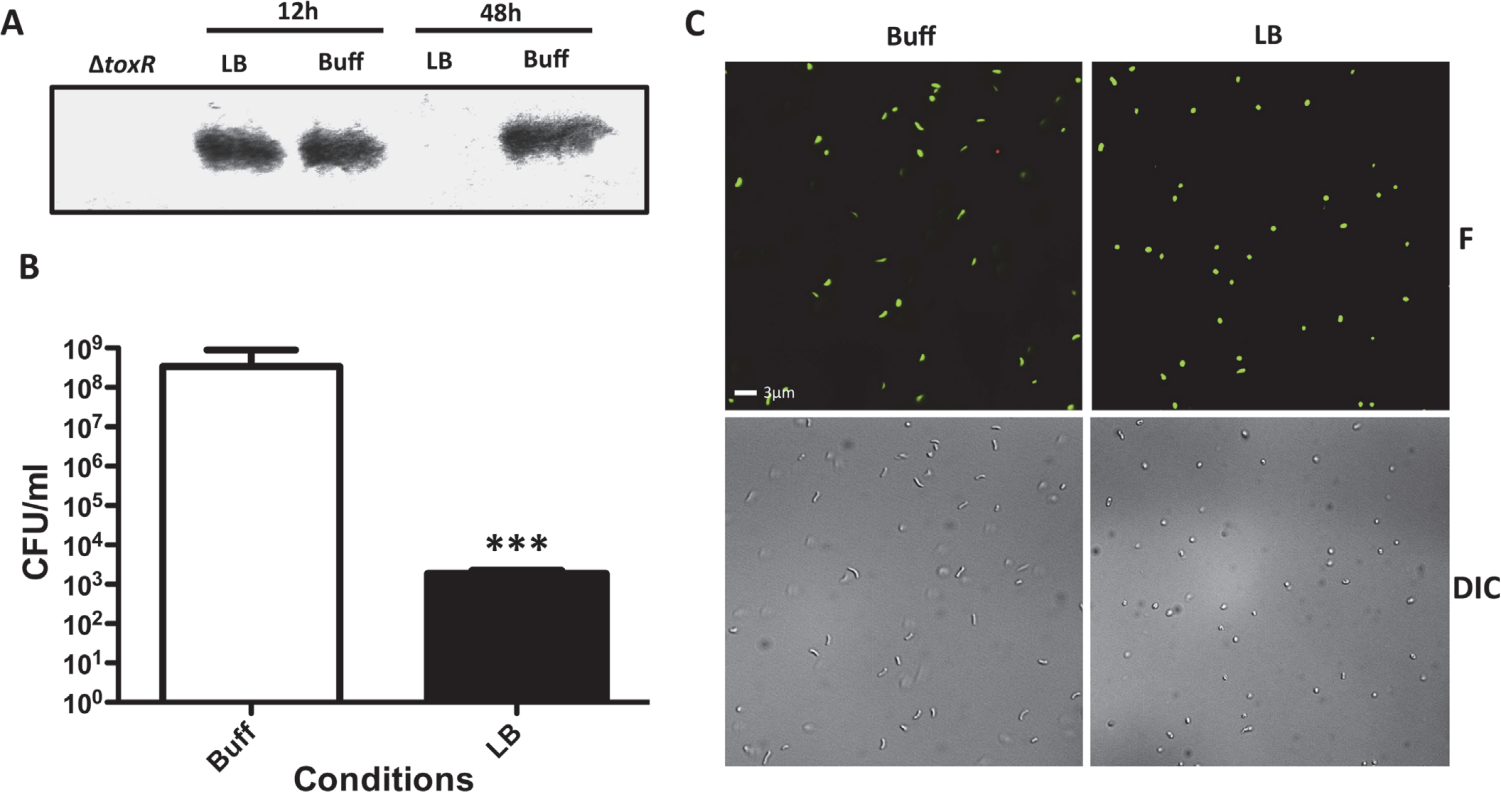
Effect of nutrient limitation in the stability of ToxR, culturability and morphology of *Vibrio parahaemolyticus*. **(A)** ToxR immunoblot of *V*. *parahaemolyticus* RIMD2210633 wild-type or *ΔtoxR* grown for either 12 or 48 hours in LB starting pH 7.0 unbuffered (LB), or LB buffered to pH 7.0 with 100 mM HEPES (Buff) **(B)** Culturability of *V*. *parahaemolyticus* RIMD2210633 grown for 48 hours in LB starting pH 7.0 unbuffered (LB), or LB buffered to pH 7.0 with 100 mM HEPES (Buff). The bars represent the mean of four independent experiments and the error bars indicate the standard deviation. Statistical comparisons were made using the student’s *t*-test and compare samples relative to 48h Buff. ***P < 0.0005. **(C)** Fluorescent (F) and differential interference contrast (DIC) images of *V*. *parahaemolyticus* RIMD2210633 grown for 48 hours under conditions as in (B). The cells were observed after treatment with the LIVE/DEAD BacLight Bacterial Viability and Counting Kit. Viable and culturable cells appear green and elongated; viable but dormant cells appear green and round; dead cells appear red and round.

*V*. *parahaemolyticus* is an intestinal pathogen that causes bloody diarrhea and is generally transmitted through the consumption of raw or uncooked fish. Recently, Whitaker *et al* showed that ToxR is required for intestinal colonization of *V*. *parahaemolyticus* in the adult murine model [56]. *V*. *parahaemolyticus* is known to enter VBNC under starvation conditions [57]. We determined whether the entry of *V*. *parahaemolyticus* into dormancy was also associated with loss of ToxR. Unexpectedly, *V*. *parahaemolyticus* cannot grow on LB with a starting pH of 9.3, however, like *V*. *cholerae*, it also alkalinizes the pH of the media during growth. Nonetheless, we found that ToxR undergoes proteolysis in *V*. *parahaemolyticus* RIMD2210633 when the bacterium is cultured on LB pH 7.0 unbuffered for 48 hours (Fig 7A). Furthermore, the bacterium loses culturability and adopts a viable coccoid form under unbuffered conditions and nutrient limitation (Fig 7B and 7C). Our data suggests that entry into dormancy might be mediated by ToxR proteolysis among other species of the family Vibrionaceae encoding ToxR.

## Discussion

The mechanisms by which *V*. *cholerae* terminates virulence and prepares for entry into the aquatic environment have recently begun to be elucidated. In this study, we found a link between the loss of virulence regulator ToxR and entry of *V*. *cholerae* into a nonculturable, dormant state. Unlike TcpP, which functions primarily in virulence gene regulation, ToxR is present in non-pathogenic strains of *V*. *cholerae* and other members of the family *Vibrionaceae*, indicating it has roles in addition to virulence. The association between the proteolysis of ToxR and entry into a dormant state sheds new light on the function of ToxR outside of the human host and the molecular mechanisms for entry into an environmentally persistent state.

We show here that ToxR in *V*. *cholerae* O395 undergoes proteolysis in late stationary phase (by 48 hours) as the medium is depleted for nutrients and becomes alkaline. That alkaline pH decreases the stability of ToxR is interesting in light of the fact that ToxR mediated activation of *ompU* expression plays a protective role for *V*. *cholerae* during organic acid stress [58]. This suggests that upregulation of ToxR repressed genes, such as *ompT*, once ToxR has been proteolyzed may protect *V*. *cholerae* under alkaline stress. The conditions identified here that induce the proteolysis of ToxR appear to be specific since we examined a wide variety of other potential stimuli and none were found to influence the stability of ToxR in *V*. *cholerae*.

The finding that RseP, the site-2 protease that influences the levels of TcpP through RIP [18], also influences the levels of ToxR in response to nutrient limitation at alkaline pH raised the possibility that ToxR might be subject to RIP. RIP is a mechanism commonly used by bacteria to rapidly adapt to changes in their environment that are important for their survival and establishment [19,20]. One of the best-studied examples of RIP activates the expression of the stress response genes associated with the σ^E^ pathway [22,23]. Other important processes regulated by RIP include sporulation [59], cell division [60], pheromone production [61], quorum sensing [62] and biofilm formation [63,64].

We also found a link between the σ^E^ dependent stress response pathway and the proteolysis of ToxR. The σ^E^ pathway has been shown to play important roles in the virulence of *V*. *cholerae* and other *Vibrio* species and is induced under alkaline pH in *Yersinia pseudotuberculosis* [44,65–68]. The role of RpoE in the proteolysis of ToxR raised the possibility that RseP might not function directly as the site-2 protease of ToxR but instead might act indirectly through its ability to activate the σ^E^ pathway. To assess this, a periplasmic truncation of ToxR that would bypass the requirement for a site-1 protease, and which has been previously shown to decrease ToxR stability, was analyzed [52,69]. This truncation was found to be proteolyzed in an RseP-dependent, RpoE-independent manner, indicating that RseP plays a direct role in the proteolysis of ToxR.

We have thus far been unable to identify any other factors that play a role in the proteolysis of ToxR. Deletion of *ompU*, encoding a protein that acts as an outer membrane sensor that responds to damage induced by antimicrobial peptides and triggers activation of the σ^E^ pathway [43] did not restore the levels of ToxR in late stationary phase at alkaline pH. This led us to determine whether the σ^E^ pathway was sufficient to trigger the proteolysis of ToxR. Given that ectopic expression of *rpoE* and induction of the σ^E^ pathway are not sufficient to trigger the proteolysis of ToxR, it appears that a second pathway might work synergistically with the σ^E^ pathway in order to initiate the RIP of ToxR. Deletion of Cpx, which partially overlaps with the σ^E^ pathway did not restore wild-type levels of ToxR under TPI conditions [46,47]. RpoS, a stationary phase sigma factor, which has been shown to influence the culturability of classical biotype *V*. *cholerae* [55], did not influence the levels of ToxR in late stationary phase at alkaline pH. DegS, the site-1 protease involved in activation of the σ^E^ pathway in *E*. *coli*, also did not influence the proteolysis of ToxR, nor did the σ^E^-regulated proteases DegP or VC0554 (S1 Table) [70].

It seemed that the proteolysis of ToxR under nutrient limitation at alkaline pH might provide an advantage to *V*. *cholerae* by increasing its ability to survive in nutrient depleted environments outside of the host. Bacteria have evolved a variety of adaptive responses that allow them to survive when conditions are not conducive to active growth. One such response is their ability to enter a dormant state where they remain viable, but are no longer culturable [71,72]. We found that under nutrient limitation at alkaline pH *V*. *cholerae* becomes nonculturable, and this occurs as the levels of ToxR are reduced. The cells remained viable as they became nonculturable, consistent with their entry into a dormant state [53]. Nutrient limitation, along with other factors such as temperature or salinity, have been previously shown to influence entry of *V*. *cholerae* into VBNC [51,72–74]. To our knowledge this is the first time that alkaline pH has been found to affect the entry of *V*. *cholerae* or other member of the Vibrionaceae into a dormant state.

From our data it can be gleaned that *V*. *cholerae* has evolved a tightly regulated response to avoid premature proteolysis of ToxR. As our results show, the presence of both nutrient limitation and alkaline pH are required to initiate the proteolysis of ToxR and the changes associated with it. Furthermore, short-term exposure to these conditions does not immediately trigger the proteolytic cascade of ToxR, indicating that the cells possess mechanisms that prevent untimely proteolysis of the virulence regulator. Given the drastic metabolic changes associated with entry into dormancy, it is possible that cells avoid proteolysis of ToxR when nutrient limitation and alkaline conditions are only transient.

We found a time difference in the proteolysis of ToxR between *V*. *cholerae* N16961 and O395. While ToxR cannot be detected in O395 cultures grown in LB for 48 hours at pH 9.3, ToxR can still be detected, even though at significantly reduced levels, in N16961 cultures under the same conditions. Nonetheless, ToxR becomes undetectable when the cultures of N16961 are incubated for a longer time. The loss of ToxR was also associated with entry into a dormant state of N16961. Our results are consistent with previous findings showing differential entry into dormancy between classical and El Tor biotypes [55]. In the aquatic environment, a significant proportion of toxigenic *V*. *cholerae* can be found as VBNC [30–32]. Thus, in the wild-type, the proteolysis of ToxR is associated with the formation of a dormant state that appears similar to the VBNC/CVEC state that is observed in the natural environment. Further work is needed to determine how *V*. *cholerae* can be recovered from this dormant state and whether this recovery is associated with the presence of ToxR.

*V*. *parahaemolyticus*, an intestinal pathogen that causes a bloody diarrhea, is known to enter VBNC under starvation conditions [57]. Interestingly, ToxR is also required for intestinal colonization of this pathogen [56]. We found that ToxR undergoes proteolysis in *V*. *parahaemolyticus* under alkaline conditions and nutrient limitation. Furthermore, similar to *V*. *cholerae*, the bacterium also loses culturability and adopts a viable coccoid form under these conditions. ToxR is encoded among other members of the family Vibrionaceae [75–78]. Our data suggests that the association between the loss of ToxR and entry into VBNC might be a widespread phenomenon among those species. In *Photobacterium profundum* ToxR becomes undetectable by immunoblot when cultured at high pressure (272 atm) relative to atmospheric pressure (1 atm) [79]. It has been suggested that ToxR is proteolyzed under high pressure and its loss may serve to increase the production of the porin OmpH (analogous to that of OmpT), which has a larger channel than OmpL (analogous to OmpU) [79,80]. This trait could be important in the deep-sea where nutrients are particularly scarce [79,80]. Furthermore, strains of *P*. *profundum* with mutations in an *rpoE*-like locus are pressure sensitive, suggesting that RpoE might also play a role in the proteolysis of ToxR in this species [81].

It remains to be determined at which stage of the *V*. *cholerae* life cycle ToxR undergoes proteolysis. It is tempting to speculate that the proteolysis of ToxR occurs during the late stages of infection, as a consequence of the resulting nutrient limitation and alkaline pH in the intestine [27,29,82]. The proteolysis of ToxR at this stage in the life cycle of *V*. *cholerae* would contribute to the termination of virulence and upregulate genes repressed by ToxR, such as *ompT*, that play a role in environmental survival. Consistent with this hypothesis is the finding that several genes involved in glycerol metabolism, which are downregulated by ToxR [35], have been found to play a role in survival in pond water [27]. Unlike ToxR, proteolysis of TcpP does not appear to be involved in the entry into VBNC since cultures transferred from inducing to non-inducing conditions, which triggers proteolysis of TcpP, do not lose culturability and keep growing in the media they are transferred to (S5 Fig).

**Fig 8:**
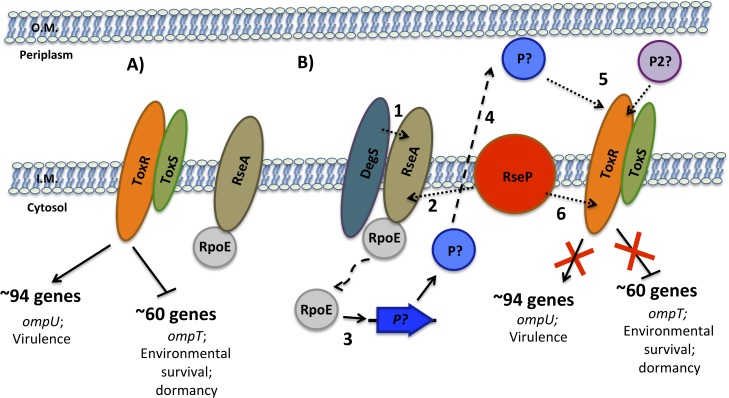
Sequential model for the RIP of ToxR during late stationary phase. **(A)** In the early stages of colonization, when nutrients are abundant, ToxRS upregulate the expression of genes such as *ompU* important for growth under these conditions and downregulate the expression of genes such as *ompT* with roles in environmental survival. **(B)** During the late stages of colonization, as nutrients become depleted and the environment becomes alkalinized, ToxR is proteolyzed. This occurs due to activation of the σ^E^ pathway via sequential degradation of RseA by either DegS or another site-1 protease (1) and RseP (2), which releases RpoE to activate its regulated genes (3). One of these genes may encode a site-1 protease (P) that enters the periplasm (4) and cleaves a periplasmic portion of ToxR, however, at least a second protease/system (P2) appears to be necessary for the site-1 proteolytic event to occur (5). This event is followed by proteolysis of an inner-membrane site of ToxR by RseP (6), which then induces and prevents, respectively, expression of ToxR repressed and activated genes.

A sequential model for the RIP of ToxR during late stationary phase is shown in Fig 8. In the early stages of colonization, when nutrients are abundant, ToxR upregulates the expression of genes such as *ompU* and those involved in virulence and downregulates the expression of genes such as *ompT* with roles in environmental survival. During the late stages of colonization, as nutrients become depleted and the environment becomes alkalinized, ToxR is proteolyzed. This occurs due to activation of the σ^E^ pathway via sequential degradation of RseA by either DegS or another site-1 protease and RseP, the site-2 protease, which releases RpoE to activate its regulated genes. It is possible that one of these genes may encode a site-1 protease that cleaves a periplasmic portion of ToxR, however, at least a second protease/system appears to be necessary for the site-1 proteolytic event to occur. Next, RseP cleaves at an intramembrane site within ToxR leading to full proteolysis of the regulator. This process induces and prevents, respectively, expression of ToxR repressed and activated genes, providing an advantage in the environment. The loss of ToxR ultimately leads to entry into dormancy.

This study has identified a RIP cascade involving RseP and RpoE that is responsible for the proteolysis of ToxR under nutrient limitation at alkaline pH. Further work is necessary in order to fully understand the pathway. This includes the identification of the site-1 protease/s that cleave ToxR as well as other genes regulated by ToxR that provide an advantage under this condition.

## Materials and Methods

### Bacterial strains, plasmids and culture conditions

*V*. *cholerae* O395 (O1 classical), *V*. *cholerae* N16961 (O1 El Tor), and *V*. *parahaemolyticus* RIMD2210633 were the wild-type strains for this study. *E*. *coli* S17-1λpir [83] was used for both cloning purposes and conjugation with *V*. *cholerae*. Unless otherwise indicated, cultures were grown O/N in LB at 37°C on a rotary shaker. To induce expression of *V*. *cholerae* virulence genes, cultures were grown in LB pH 6.5 at 30**°**C. Antibiotics were used at the following concentrations: ampicillin (Ap), 100 μg/ml; gentamicin (Gm), 30 μg/ml; streptomycin (Sm), 1 mg/ml.

### Mutant construction

Deletion and substitution mutations were constructed by PCR amplifying two approximately 500 bp fragments of DNA upstream and downstream of the region of interest. For the substitutions, nucleotide changes were introduced into the primers. After amplification, the inserts were digested with the appropriate restriction enzymes, ligated into pKAS32 [84] and electroporated into *E*. *coli* S17-1λ*pir* [83]. The various mutations were then transferred into *V*. *cholerae* by allelic exchange [84]. *V*. *cholerae* classical biotype contained plasmid pMIN1 [85] conferring Gm^r^ as a counter-selection for conjugation. DNA sequencing was used to confirm the correct deletion or mutant sequence in the *V*. *cholerae* genome. The sequences of the primers used in this study are available upon request.

### Protein electrophoresis and western blotting

Whole cell protein extracts were prepared from cultures grown in LB pH 6.5 at 30°C. Protein concentration was quantitated using Pierce BSA Protein Assay quantitation kit from Thermo Scientific. Protein samples were normalized and an equal amount of protein was loaded per well. The extracts were subjected to SDS-PAGE on 16% Tris Glycine gels (Invitrogen) and transferred to nitrocellulose using iBlot (Invitrogen). The membranes were blocked O/N in Tris-Buffered Saline, 3% BSA. Primary antibodies were diluted 1:10,000 in TBST (Tris-Buffered Saline, 0.5% Tween-20). Membranes were incubated with primary antibody for 2 hours at room temperature. After incubation, the membranes were washed with TBST four times. Goat anti-rabbit secondary antibodies (BioRad) were diluted 1:10,000 in TBST and incubated for 30 minutes at room temperature. The membranes were washed 4 times with TBS (Tris-Buffered Saline). Reactive protein bands were detected via ECL (Amersham).

### Fluorescent microscopy

From each bacterial suspension, a 1 ml aliquot was centrifuged at 10,000 rpm for 1 min and the pellet was resuspended in phosphate buffered saline (PBS) twice. 1 ml of the mixture was then transferred to a 50 ml centrifuge tube with 24 ml PBS, and was centrifuged at 7,840 rpm for 10 minutes. The pellet was resuspended in 10 ml PBS, and a 1 ml aliquot was stained with a 3 μl mixture (1:1) of SYTO9 and propidium iodide (PI) nucleic acid stain (Molecular Probes, OR). After incubation in the dark for 15 min at 25°C, the stained cells were mounted on a glass slide and low-fluorescence immersion oil was added on the cover slide. The cells were then examined with a Zeiss Ax-iovert inverted microscope, and pictures were taken with AxioVision microscopy software (Zeiss).

### CFU counts

Samples were serially diluted and 5 μl of each dilution was plated on LB. Plates were incubated overnight at 37 °C, and colony forming units were counted. 1 ml of initial culture was plated for those strains where no colonies were recovered after plating 5 μl. Values were plotted using Prism software. The bars represent the mean of at least four independent experiments and the error bars indicate the standard deviation.

## Supporting Information

S1 Fig Stability of GbpA under TPI conditions and effect of RpoE in the proteolysis of ToxR. **(A)** GbpA immunoblot of *V*. *cholerae* O395 wild-type after 12 hours and 48 hours of growth in LB: LB pH 7.0 with 100 mM HEPES (Buff), LB starting pH 9.3 unbuffered (pH 9.3). **(B)** ToxR immunoblot of *V*. *cholerae* O395 wild-type (WT) and *ΔrseP* strains carrying an expression vector (pBAD22) encoding the gene for RpoE. Expression of *rpoE* was induced after addition of 0.1mg/ml arabinose (Ara) to overnight cultures of the strains. Whole cell protein was extracted at different time points after addition of Ara. **(C)** ToxR immunoblot of a wild-type (WT) strain after growth in LB regular (LB), LB 3% ethanol (3% EtOH), after exposure to P2 peptide for 1 hour (P2 peptide).
(TIF)

S2 Fig Culturability of growth *V*. *cholerae* wild-type and *ΔtoxR* over time. **(A)** CFU/ml of O395 *ΔtoxR* strain grown at different time points in LB: LB pH 7.0 with 100 mM HEPES (Buff), LB starting pH 9.3 unbuffered (pH 9.3). The bars represent the mean of four independent experiments and the error bars indicate the standard deviation. Statistical comparisons were made using the student’s *t*-test and compare samples relative to Δ*toxR* 12h Buff. ***P < 0.0005. **(B)** Growth curve of *V*. *cholerae* O395 wild-type and Δ*toxR* in LB: LB pH 7.0 with 100 mM HEPES (Buff), LB starting pH 9.3 unbuffered (pH 9.3). Each data point represents the mean of three experiments and the error bars correspond to the standard deviation.
(TIFF)

S3 Fig Culturability of *V*. *cholerae* strains over time on PBS and *toxR-phoA* stability. **(A)** CFU/ml of O395 wild-type strain grown at different time points in PBS: PBS pH 7.0 with 100 mM HEPES (Buff), PBS starting pH 9.3 unbuffered (pH 9.3). The bars represent the mean of four independent experiments and the error bars indicate the standard deviation. Statistical comparisons were made using the student’s *t*-test and compare samples relative to wild-type 0h Buff. **P < 0.005, ***P < 0.0005. **(B)** CFU/ml of O395 Δ*toxR* strain grown at different time points in PBS: PBS pH 7.0 with 100 mM HEPES (Buff), PBS starting pH 9.3 unbuffered (pH 9.3). The bars represent the mean of four independent experiments and the error bars indicate the standard deviation. Statistical comparisons were made using the student’s *t*-test and compare samples relative to Δ*toxR* 0h Buff. *P < 0.05, ***P < 0.0005. **(C)** ToxR immunoblot of wild-type (WT) and *toxR-phoA* strains after 48 hours of growth in LB: LB pH 7.0 with 100 mM HEPES (Buff), LB starting pH 9.3 unbuffered (pH 9.3).
(TIFF)

S4 Fig Proteolysis of ToxR during late stationary phase at alkaline pH in the El Tor biotype. **(A)** ToxR immunoblot of N16961 wild-type or Δ*toxR* grown for either 12, 48 or 72 hours in: LB starting pH 9.3 unbuffered (pH 9.3), or LB buffered to pH 7.0 with 100 mM HEPES (Buff). **(B)** Culturability of N16961 wild-type after 72 hours in: LB starting pH 9.3 unbuffered (pH 9.3), or LB buffered to pH 7.0 with 100 mM HEPES (Buff). The bars represent the mean of four independent experiments and the error bars indicate the standard deviation. Statistical comparisons were made using the student’s *t*-test and compare samples relative to wild-type 72h Buff. ***P < 0.0005. **(C)** Morphology and viability of N16961 after 72 hours as in (B). The cells were observed with fluorescence microscopy and differential interference contrast (DIC) after treatment with the LIVE/DEAD BacLight Bacterial Viability and Counting Kit. Viable and culturable cells appear green and elongated; viable but dormant cells appear green and round; dead cells appear red and round.
(TIFF)

S5 Fig Culturability of *V*. *cholerae* after transfer from inducing to non-inducing conditions. *V*. *cholerae* O395 wild-type was grown overnight under inducing conditions (LB starting pH 6.5, 30°C). The cultures were then transferred to non-inducing conditions (LB, 37°C) and CFU/ml of cultures was measured at different time points. The bars represent the mean of three independent experiments and the error bars indicate the standard deviation.
(TIFF)

S1 Table Stability of ToxR in *V*. *cholerae* strains. *V*. *cholerae* O395 strains were grown in LB pH 9.3 unbuffered for 12 or 48 hours. Total protein was extracted from the cultures and the presence of ToxR was determined through immunoblots. **+**, ToxR was detectable.-, ToxR was not detectable.
(TIFF)
