# Supplementary figures and images for: Proteolysis of Virulence Regulator ToxR Is Associated with Entry of Vibrio cholerae into a Dormant State
Source: PLoS Genet. 2015 Apr 7;11(4):e1005145. doi: 10.1371/journal.pgen.1005145 (PMC4388833; doi:10.1371/journal.pgen.1005145)

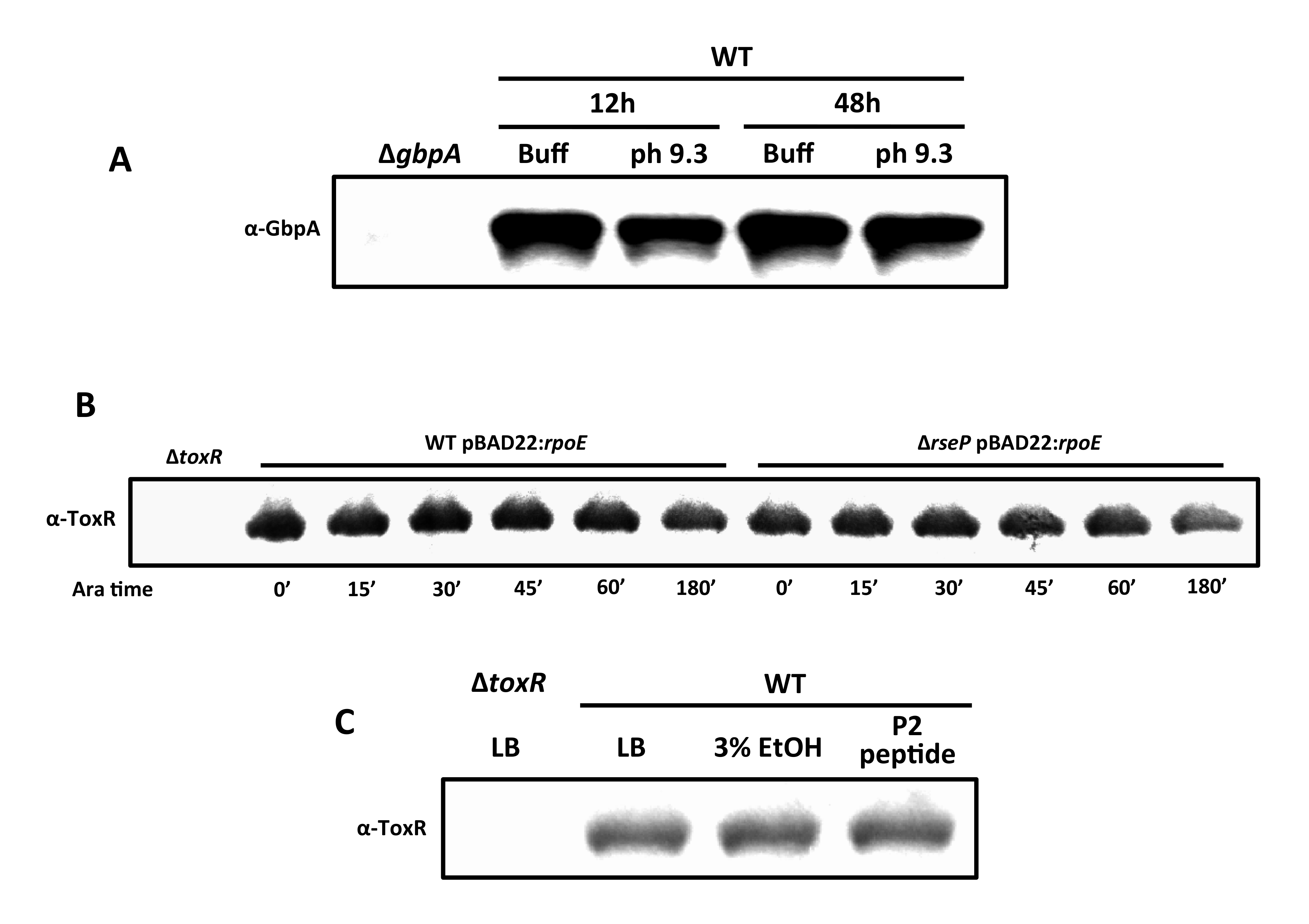

Supplement: S1 Fig — (A) GbpA immunoblot of V. cholerae O395 wild-type after 12 hours and 48 hours of growth in LB: LB pH 7.0 with 100 mM HEPES (Buff), LB starting pH 9.3 unbuffered (pH 9.3). (B) ToxR immunoblot of V. cholerae O395 wild-type (WT) and ΔrseP strains carrying an expression vector (pBAD22) encoding the gene for RpoE. Expression of rpoE was induced after addition of 0.1mg/ml arabinose (Ara) to overnight cultures of the strains. Whole cell protein was extracted at different time points after addition of Ara. (C) ToxR immunoblot of a wild-type (WT) strain after growth in LB regular (LB), LB 3% ethanol (3% EtOH), after exposure to P2 peptide for 1 hour (P2 peptide). (TIF) [file pgen.1005145.s001.tif]

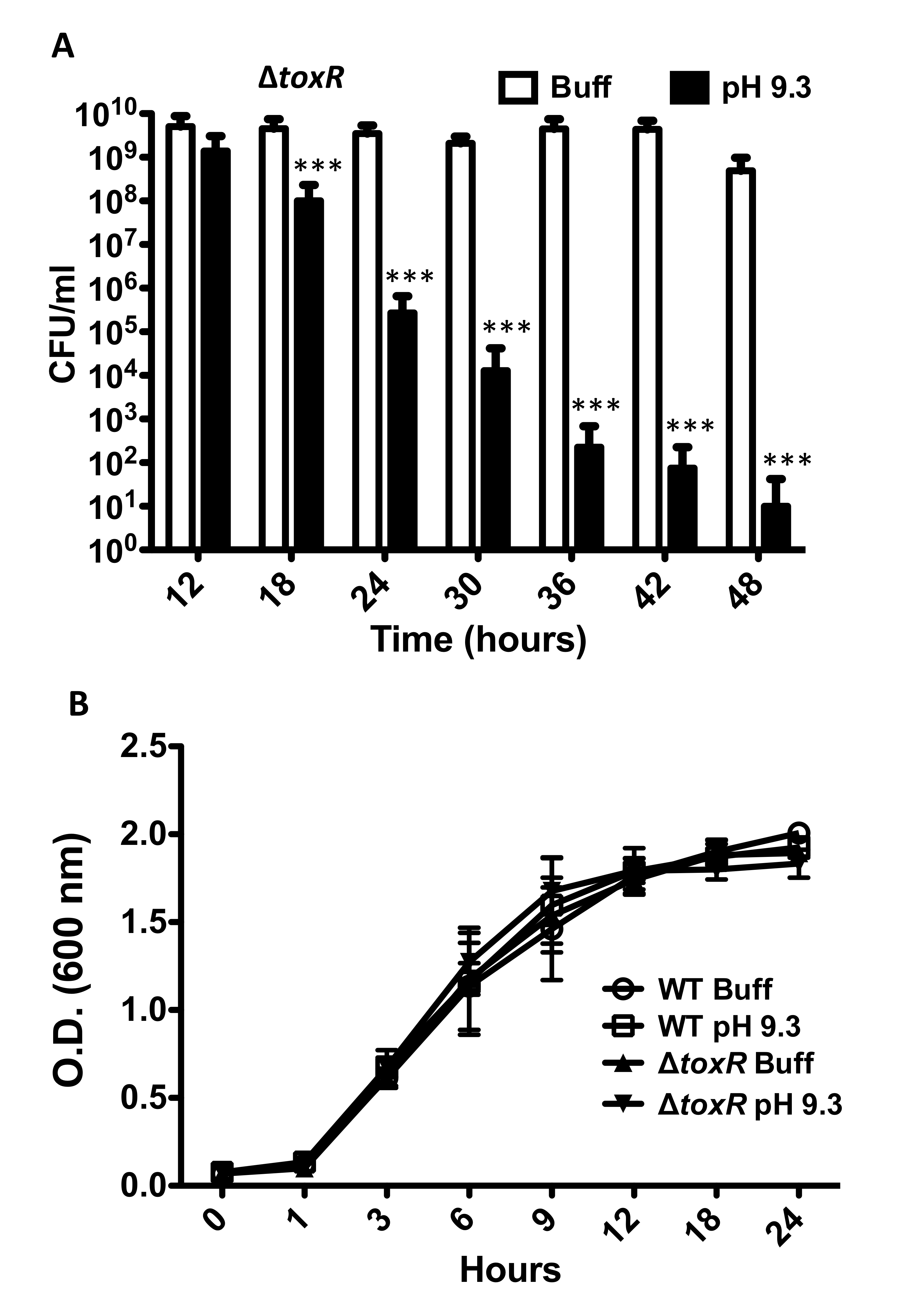

Supplement: S2 Fig — (A) CFU/ml of O395 ΔtoxR strain grown at different time points in LB: LB pH 7.0 with 100 mM HEPES (Buff), LB starting pH 9.3 unbuffered (pH 9.3). The bars represent the mean of four independent experiments and the error bars indicate the standard deviation. Statistical comparisons were made using the student’s t-test and compare samples relative to ΔtoxR 12h Buff. ***P < 0.0005. (B) Growth curve of V. cholerae O395 wild-type and ΔtoxR in LB: LB pH 7.0 with 100 mM HEPES (Buff), LB starting pH 9.3 unbuffered (pH 9.3). Each data point represents the mean of three experiments and the error bars correspond to the standard deviation. (TIFF) [file pgen.1005145.s002.tiff]

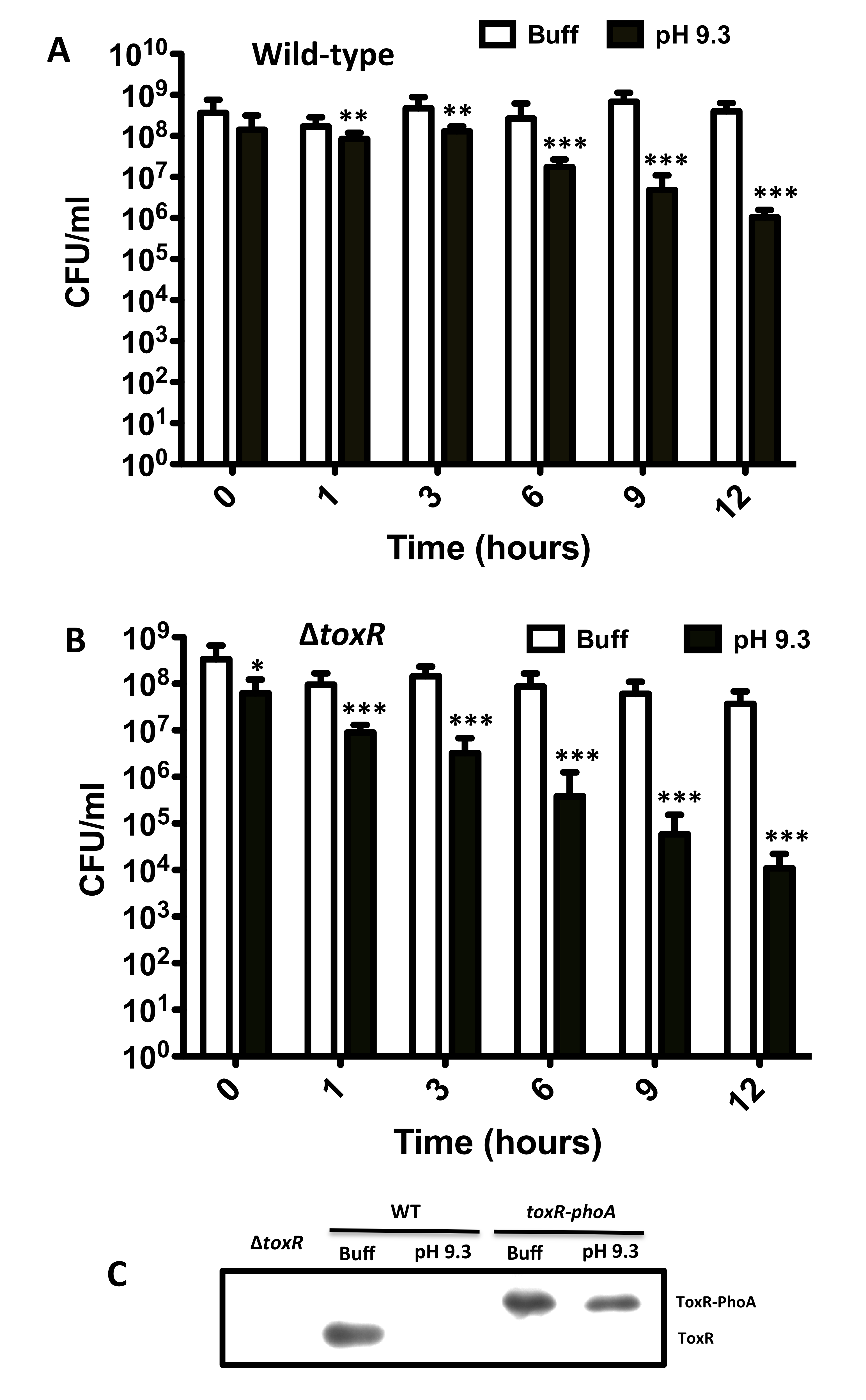

Supplement: S3 Fig — (A) CFU/ml of O395 wild-type strain grown at different time points in PBS: PBS pH 7.0 with 100 mM HEPES (Buff), PBS starting pH 9.3 unbuffered (pH 9.3). The bars represent the mean of four independent experiments and the error bars indicate the standard deviation. Statistical comparisons were made using the student’s t-test and compare samples relative to wild-type 0h Buff. **P < 0.005, ***P < 0.0005. (B) CFU/ml of O395 ΔtoxR strain grown at different time points in PBS: PBS pH 7.0 with 100 mM HEPES (Buff), PBS starting pH 9.3 unbuffered (pH 9.3). The bars represent the mean of four independent experiments and the error bars indicate the standard deviation. Statistical comparisons were made using the student’s t-test and compare samples relative to ΔtoxR 0h Buff. *P < 0.05, ***P < 0.0005. (C) ToxR immunoblot of wild-type (WT) and toxR-phoA strains after 48 hours of growth in LB: LB pH 7.0 with 100 mM HEPES (Buff), LB starting pH 9.3 unbuffered (pH 9.3). (TIFF) [file pgen.1005145.s003.tiff]

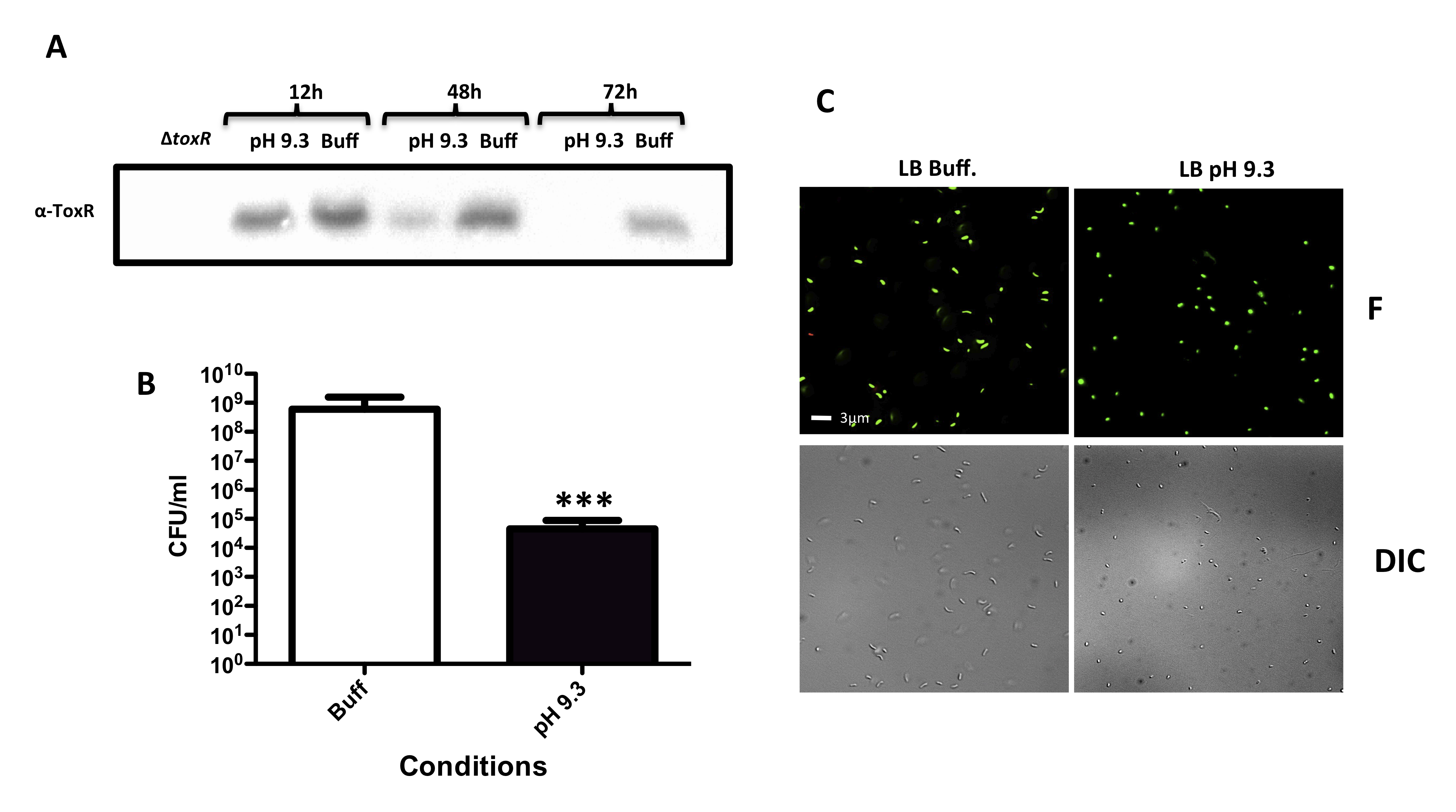

Supplement: S4 Fig — (A) ToxR immunoblot of N16961 wild-type or ΔtoxR grown for either 12, 48 or 72 hours in: LB starting pH 9.3 unbuffered (pH 9.3), or LB buffered to pH 7.0 with 100 mM HEPES (Buff). (B) Culturability of N16961 wild-type after 72 hours in: LB starting pH 9.3 unbuffered (pH 9.3), or LB buffered to pH 7.0 with 100 mM HEPES (Buff). The bars represent the mean of four independent experiments and the error bars indicate the standard deviation. Statistical comparisons were made using the student’s t-test and compare samples relative to wild-type 72h Buff. ***P < 0.0005. (C) Morphology and viability of N16961 after 72 hours as in (B). The cells were observed with fluorescence microscopy and differential interference contrast (DIC) after treatment with the LIVE/DEAD BacLight Bacterial Viability and Counting Kit. Viable and culturable cells appear green and elongated; viable but dormant cells appear green and round; dead cells appear red and round. (TIFF) [file pgen.1005145.s004.tiff]

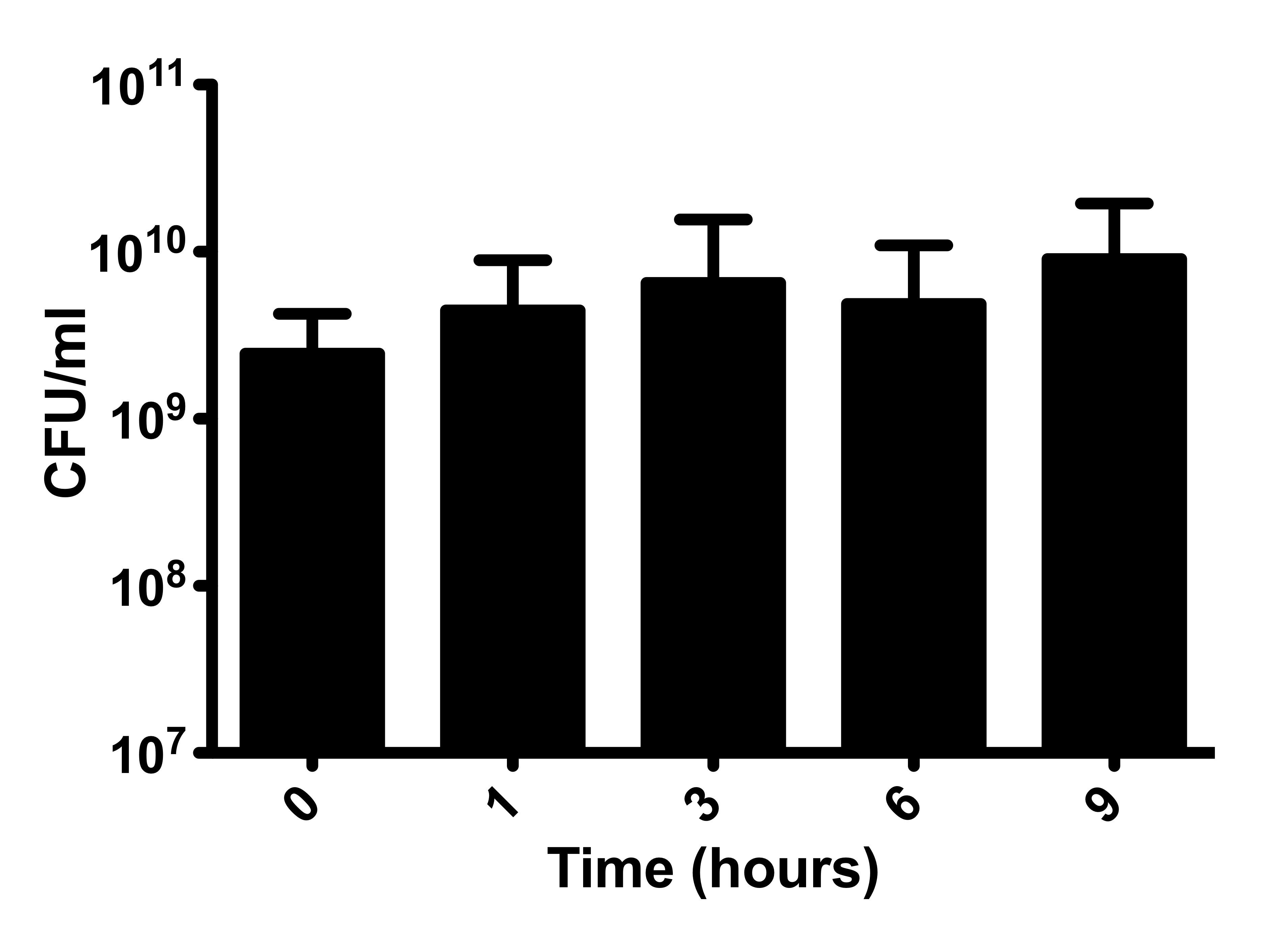

Supplement: S5 Fig — V. cholerae O395 wild-type was grown overnight under inducing conditions (LB starting pH 6.5, 30°C). The cultures were then transferred to non-inducing conditions (LB, 37°C) and CFU/ml of cultures was measured at different time points. The bars represent the mean of three independent experiments and the error bars indicate the standard deviation. (TIFF) [file pgen.1005145.s005.tiff]

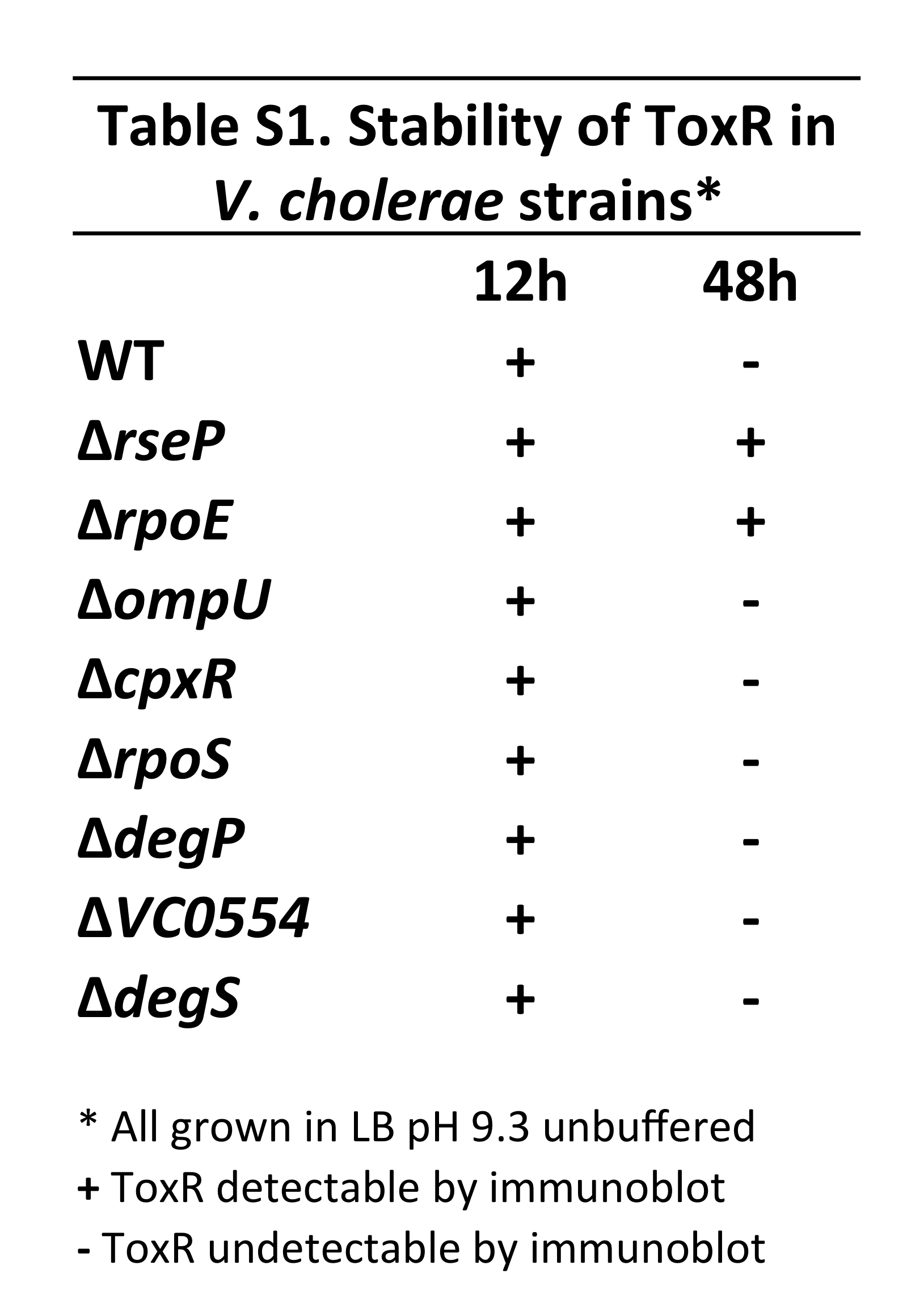

Supplement: S1 Table — V. cholerae O395 strains were grown in LB pH 9.3 unbuffered for 12 or 48 hours. Total protein was extracted from the cultures and the presence of ToxR was determined through immunoblots. +, ToxR was detectable.-, ToxR was not detectable. (TIFF) [file pgen.1005145.s006.tiff]
